# Supplementary material for: Dental Epithelial Stem Cells as a Source for Mammary Gland Regeneration and Milk Producing Cells In Vivo
Source: Cells. 2019 Oct 22;8(10):1302. doi: 10.3390/cells8101302 (PMC6830078; doi:10.3390/cells8101302)
Supplement: Supplementary file 1 [file cells-08-01302-s001.pdf]

Supplementary figures

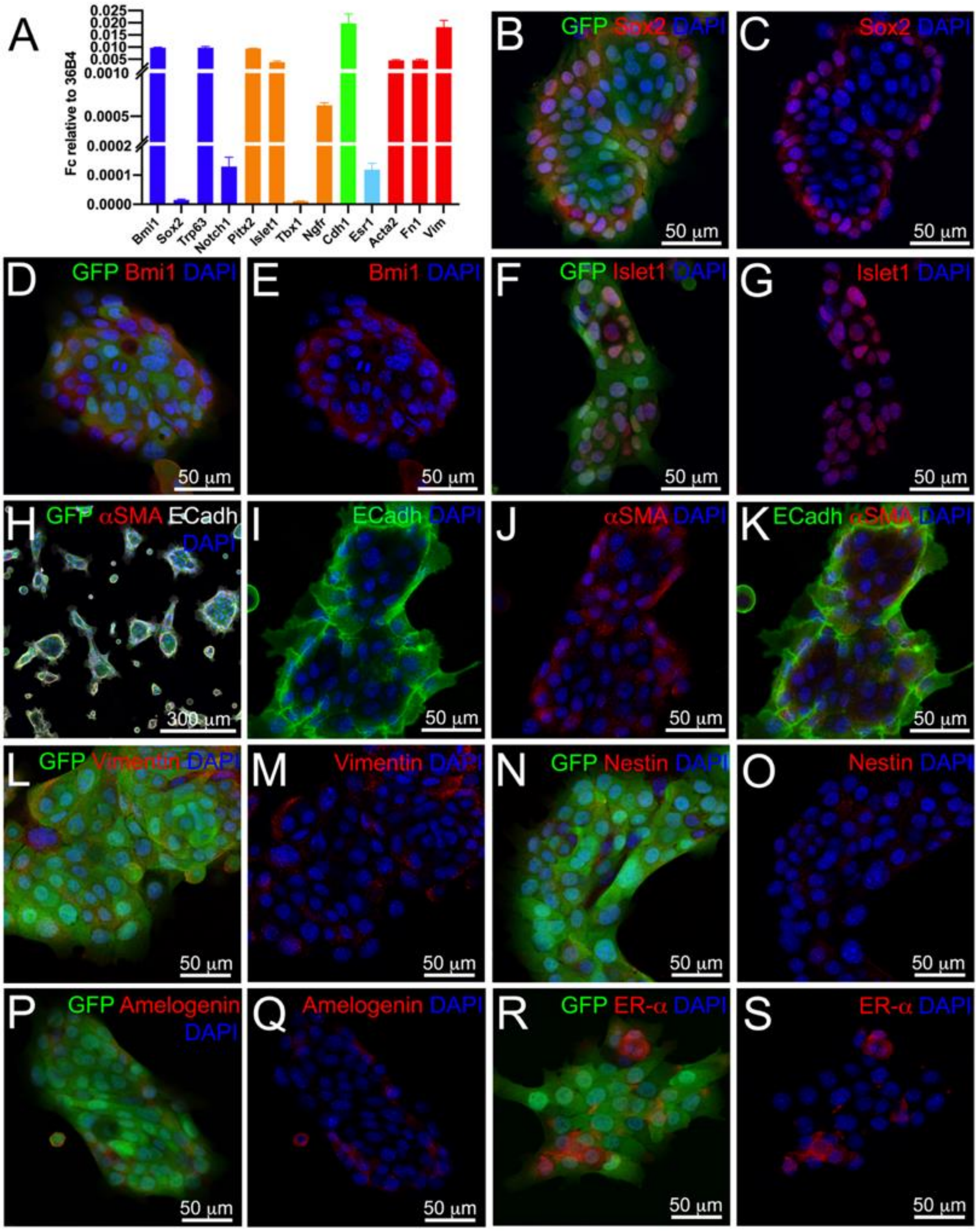

Figure S1. Characterization of DESCs. **A)** Real time PCR analysis of expression of genes coding for DESCs markers, epithelial markers, and mesenchymal markers. B**-S)** Immunofluorescent staining showing expression of stem cell markers (B-E), classical incisor's markers (F, G), epithelial vs mesenchymal markers (H-O), the ameloblastic differentiation marker amelogenin (P, Q), and estrogen receptor α (R, S). Abbreviations: ER- α, estrogen receptor α ; GFP, green fluorescent protein.

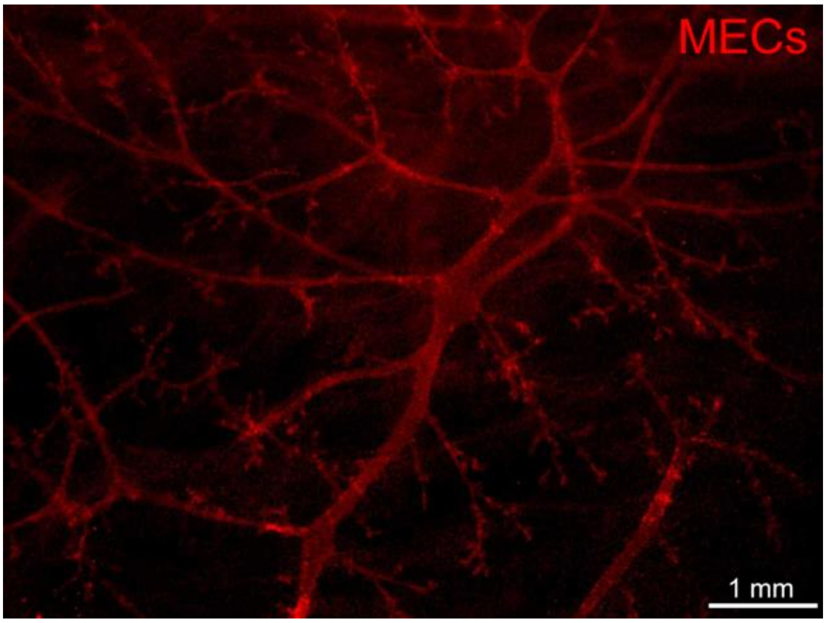

Figure S2. Positive control of mammary reconstitution: dsRed-expressing mammary epithelial cells (MECs) injected alone in cleared fat pads can reconstitute a fully developed mammary epithelium. Abbreviations: MECs, mammary epithelial cells.

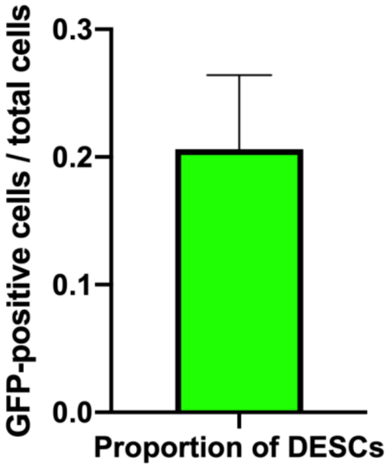

Figure S3. Proportion of DESCs-derived GFP-positive cells contributing to mammary ducts upon transplantation with MECs (1:1 ratio).

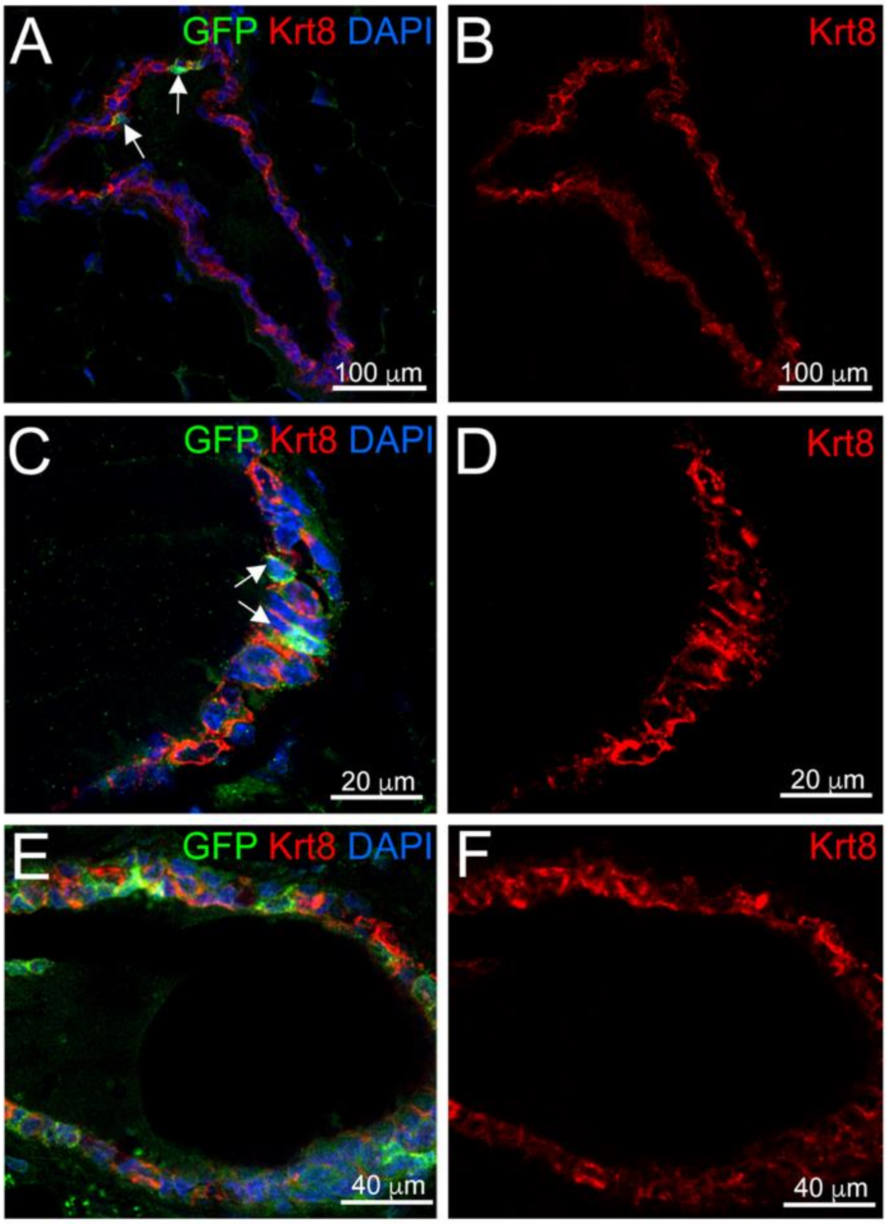

Figure S4. DESCs integrate in the luminal compartment of the regenerated mammary epithelium (white arrowheads). A-D) Immunofluorescent staining against GFP (green color) and Keratin 8 (red color) on mammary fat pads injected with a 1:1 mixture of DESCs and MECs. B and D show the single red channel (Krt8) of A and C, respectively. E, F) Immunofluorescent staining against GFP (green color) and Keratin 8 (red color) on mammary fat pads injected with DESCs. F shows single red channel (Krt8) of E. Abbreviations: GFP, green fluorescent protein; Krt8, keratin 8.

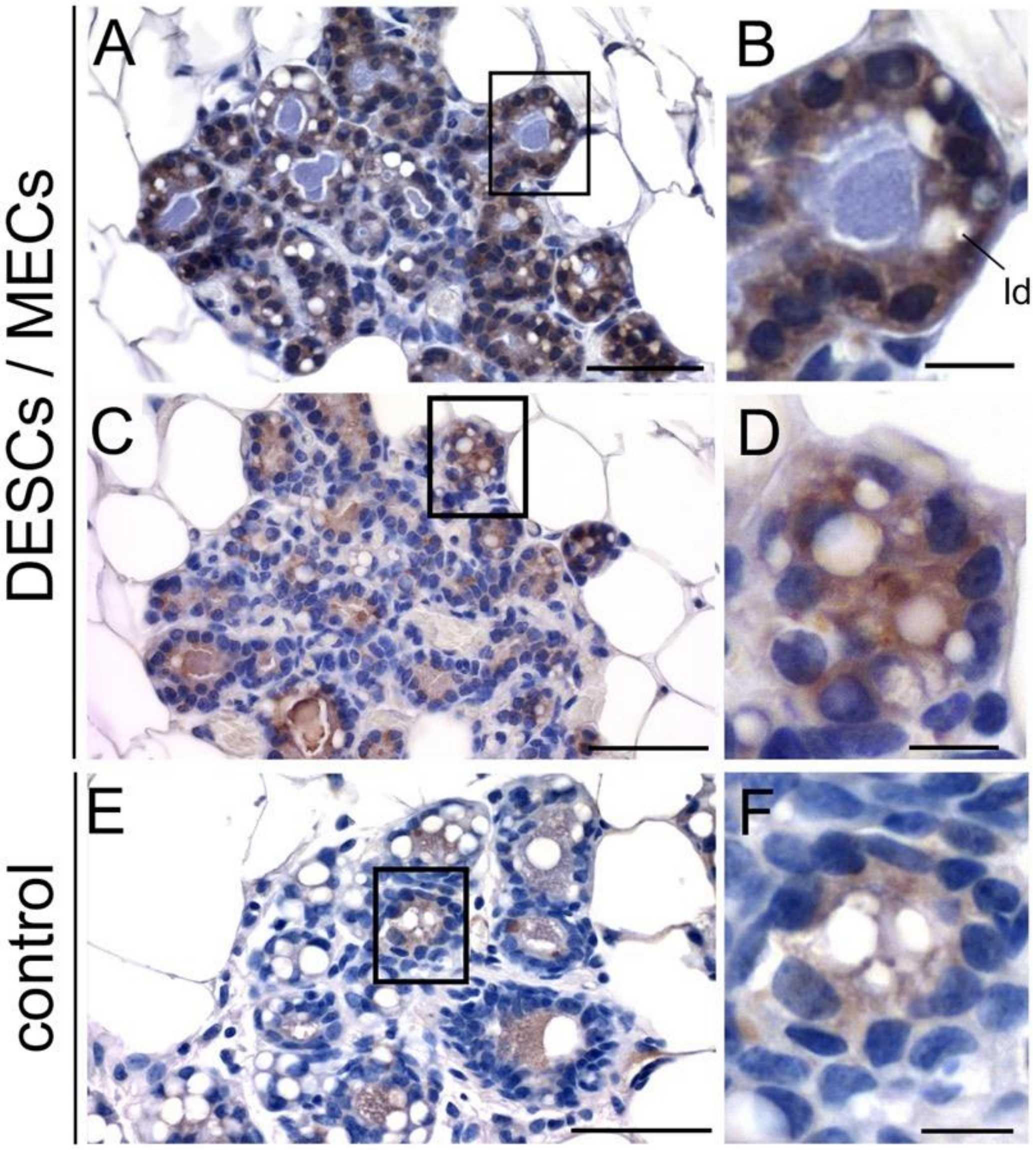

Figure S5. DESCs give rise to milk-producing, β-casein-positive cells. A-D) Immunohistochemistry against GFP (A, B) and β–casein (C, D) on mammary fat pads injected with a 1:1 mixture of DESCs and MECs. E, F) Control immunohistochemistry against β-casein on a P16 (pregnancy day 16) mammary gland. Scale bars: A, C, E: 40 µm; B, D, F: 10 µm.

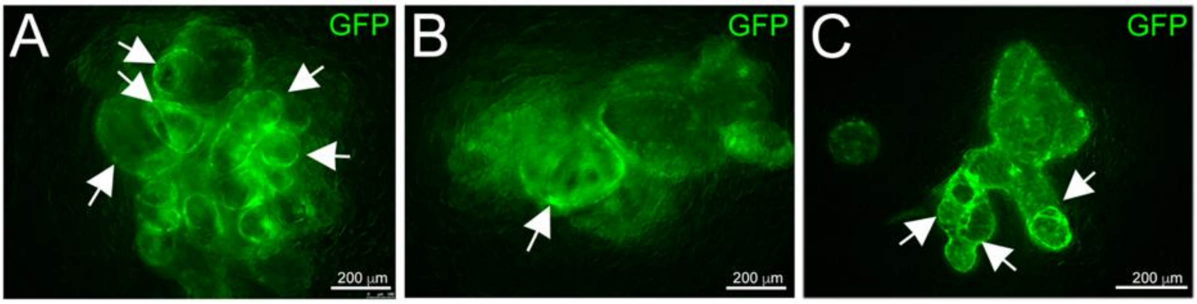

Figure S6. DESCs transplanted in cleared mammary fat pads give rise to rudimentary branched structures. A-C) Whole mount imaging of mammary fat pads transplanted with GFP-positive DESCs. White arrows indicate some of the observed branches.
